# Regulation of podocyte lesions in diabetic nephropathy via miR-34a in the Notch signaling pathway

**DOI:** 10.1097/MD.0000000000005050

**Published:** 2016-11-04

**Authors:** Xiangying Zhang, Shuping Song, Huixin Luo

**Affiliations:** Department of Endocrinology, Tianjin Hospital, Tianjin, P.R. China.

**Keywords:** diabetic nephropathy, high glucose, miR-34a, Notch signaling pathway, podocyte lesions

## Abstract

**Background:**

The activation of the Notch signaling pathway has been shown to play an important role in diabetic nephropathy (DN) development. Besides, Notch-1 is a target gene in miR-34a. However, the regulation of the podocyte lesions involved in DN by miR-34a has not been identified.

**Methods:**

This study utilized miR-34a mimics and small interfering RNA transfection to construct miR-34a overexpression and lower-expression model to investigate the effect of miR-34a on the regulation of the Notch signaling pathway and podocyte lesions in DN. Western blotting and real-time quantitative polymerase chain reaction were applied for the quantitative testing of mRNA and protein expression. Apoptosis of podocyte was detected by TUNEL staining.

**Results:**

In high-glucose (HG) conditions, miR-34a overexpression inhibited the expression of Notch 1, Jagged 1, NICD, Hes 1, and Hey 1 proteins. Further, cleaved caspase-3, Bax, and phosphorylation of p53 (p-p53) were reduced significantly. Therefore, miR-34a overexpression inhibited the Notch signaling pathway and podocyte lesions induced by HG. β-arrestin was slightly reduced in HG conditions. Meanwhile, miR-34a overexpression could remit the inhibition.

**Conclusion:**

Results from this study provide evidence that miR-34a may offer a new approach for the treatment of diabetes.

## 1 Introduction

Diabetic nephropathy (DN) is a lethal complication of diabetes with undefined etiology and limited treatment options. Previous studies have demonstrated that the pathological characteristics of DN include glomerular basement membrane (GBM) thickening, extracellular matrix (ECM) accumulation, glomerular sclerosis, renal tubular atrophy, and renal interstitial fibrosis. Studies have also found that podocyte damage plays an important role in the occurrence and development of DN. Podocytes are terminal differentiation of glomerular epithelial cells, which attach to the outside of the GBM, and are an important part of the glomerular filtration units. Further, podocytes are a highly differentiated cell with poor proliferation ability. When podocytes are damaged, slit diaphragm proteins (nephrin and podocin) are lost, and the glomerular filtration electrostatic barrier weakens, which promotes the occurrence of proteinuria. Currently, the most well-known apoptosis pathways in podocytes include Bcl-2, p53, NK-κB, and the like, which are activated when DN occurs. At the same time, the regulation function of damaged podocyte is disturbed.

The Notch protein family is composed of 4 receptors (Notch-1, Notch-2, Notch-3, Notch-4), 5 canonical Jagged-like ligands (Jag 1, Jag 2), and 3 delta-like ligands (Dll 1,Dll 3,Dll 4).^[1]^ Notch receptors (Notch 1–4) are a family of transmembrane proteins, present in most vertebrates and invertebrates and act as a key regulator of cellular development, differentiation, apoptosis, and variation. Notch receptors consist of N-terminal Notch extracellular domain (NECD) and C-terminal Notch intracellular domain (NICD). In mammals, Notch ligands include Jagged-like and Delta-like receptors based on the homology of the ligands seen in the drosophila.^[2]^ The combination of ligands and receptors change the Notch receptor conformation, and γ-secretase mediates the protein hydrolysis and release the NICD. NICD transfers into the nucleus and activates gene transcription of Hes1 and Hey1, antagonistic genes that hinder gene expression and affect cell differentiation, proliferation, and apoptosis.

MicroRNAs (miRNAs) are noncoding RNAs with approximately 18 to 24 nucleotides and are involved in various cellular processes, including cell survival, differentiation, and apoptosis. Further, they regulate gene expression by targeting the 3′-untranslated region.^[3–5]^ Additional studies show that miRNAs play an important role in podocytes^[6,7]^ and are upregulated in diabetic mice in high-glucose (HG) conditions and regulate podocyte apoptosis.^[8,9]^ Nonvisual beta-arrestins (β-arrestins) play a major role in regulation of Notch signaling and require further studies.^[10]^ The present study investigates the role of miR-34a in regulating the lesion progression of podocyte in DN via the Notch signaling pathways.

## 2 Methods

### 2.1 Cell culture

Mouse podocytes (Cell Resource Center of Peking Union Medical College, Beijing, China) were conditionally immortalized using a temperature-sensitive SV40 large T-cell antigen (tsA58 Tag). The cells were cultured in RPMI 1640 medium (containing 10% fetal bovine serum, 10 U/mL γ-interferon) at 33°C and 5% CO_2_. After passage, the cells were transferred into RPMI 1640 medium without γ-interferon at 37°C and 5% CO_2_ for 10 to 14 days. The cells were divided into 4 groups as follows: normal glucose (NG), containing 5.6 mM (control group); HG cultured in MEM medium containing 25 mM glucose; empty vector group (EVG); and miR-34a overexpression (miR-34a OE) with 25 mM glucose. Cells were exposed to the above conditions for 24 hours. The ethics approval of this study has been obtained from ethics committee.

### 2.2 Lipofectamine and siRNA transfection

MiR-34a mimics (Invitrogen, Carlsbad, CA, USA) and smal linterfering RNA (siRNA) (Santa Cruz, CA, USA) were applied to create miR-34a overexpression and lower expression in podocytes. Has-miR-34a mimics sequences as follows: forward, 5′-UGGCAGUGUCUUAGCUGGUUG-3′; reverse, 5′-ACCAGCUAAGACACUGCCAUU-3′. Ten microliters Lipofectamine 2000 was diluted with 250 μL serum-free RPMI 1640 and incubated at room temperature for 5 minutes. Plasmids were then added to the diluted solution and incubated for 20 minutes at room temperature to allow the liposome to encase the plasmid sufficiently. The complex was joined into 6-well platesand incubated for 6 hours at 37°C, 5% CO_2_. Eight microliters siRNA duplexes and 6 μL siRNA transfection reagent were added into 100 μL siRNA transfection medium and incubated 30 to 45 minutes at room temperature. Before transfection, 800 μL siRNA transfection medium was added to each tube of transfection reagent. The mixture of 1 mL transfection reagent and podocyte was then incubated at 37°C for 6 hours. Finally, the medium was changed to conventional medium and cultured sequentially for 48 hours.

### 2.3 Western blotting analysis

Podocytes were washed with precooling saline twice followed by 500 μL of pyrolysis buffer (20 mmol/L Tris-HCl, 2.5 mmol/L EDTA, 10% glycerol, 0.1% SDS, 1% Triton X-100). The buffer was placed in an icebath for 40 minutes and centrifuged 12,000/minute for 20 minutes at 4°C. The supernate was discarded. The extracted protein, SDS polyacrylamide gel electrophoresis, was transferred to the polyvinylidene fluoride membrane. The membranes were blocked by 5% nonfat milk for 2 hours at 37°C and then incubated with primary antibodies at 4°C overnight. The primary polyclonal antibodies were as follows: Jagged 1 (Santa Cruz) ‘Notch 1‘NICD 1‘Bcl-2 (1:200, rabbit; Abcam, Cambridge, UK); Hes 1, Hey 1 (1:2000, rabbit; Abcam); Bax (1:3000, rabbit; Proteintech, CA, USA); β-arrestin-1 (1:2000, rabbit; Abcam); β-arrestin-2 (1:2000, rabbit; Abcam); β-actin (1:1000, rabbit; Bioss, Beijing, China). After being washed with Tween20-Tris buffer solution, horseradish peroxidase conjugated secondary antibody (1:5000, goat) was incubated for 1 hour at 37°C. Protein bands were visualized using an ECL kit (Thermo Scientific, CA, USA). β-actin was used as an internal reference for quantification and protein levels were quantified using Image-Pro Plus 6.0 software.

### 2.4 Real-time quantitative PCR

The extracted RNA was treated with 1 mL Trizol reagent (Invitrogen). RNA concentration was measured at 260 to 280 nm and the required A260/A280 value was 1.8 to 2.0. A reverse transcription reaction system of 20 μL was employed with the SuperScript First-Stand Synthesis system (Invitrogen) to synthesize cDNA. PCR amplification was completed by ABI PRISM 7900 thermocycler using SYBR Premix Taq (Applied Biosystems). The reaction conditions were as follows: an initial denaturation step at 95°C for 4 minutes, followed by 35 cycles at 94°C for 20 seconds, 55°C for 30 seconds, 72°C for 20 seconds, 72°C for 2 minutes, and a final elongation step at 72°C for 10 minutes. The primers used for amplification were as follows: Notch 1 (Forward: 5′-GTGGATGACCTAGGCAAGTCG-3′, Reverse: 5′-GTCTCCTCCTTGTTGTTCTGC-3′); Jagged 1 (Forward: 5′-AGAAGTCAGAGTTCAGAGGCGTCC-3′, Reverse: 5′-AGTAGAAGGCTGTCACCAAGCCAAC-3′) Hes 1 (Forward: 5′-CACGACACCGGACAAACCA-3′, Reverse: 5′-GCCGGGAGCTATCTTTCTTAAGTG-3′; Hey 1 (Forward: 5′-AAGACGGAGAGGCATCATCGAG-3′, Reverse: 5′-CAGATCCCTGCTTCTCAAAGGCAC-3′); β-arrestin-1 (Forward: 5′-AAGCT CCAGT TTGCT CCTGA-3′, Reverse: 5′-TCTGT TCTTC TTGAC GCTCT-3′); β-arrestin-2 (Forward: 5′-AAGGT ACAGT TTGCT CCTGA-3′, Reverse: 5′-TCTGA TCTTC TTGAC GGTCT-3′); β-actin (Forward: 5′-TTCCTTCTTGGGTATGGAAT-3′, Reverse: 5′-GAGCAATGATCTTGATCTTC-3′). Levels of gene expression were expressed relative to β-actin and calculated using the 2^−ΔΔCt^ method.

### 2.5 TUNEL staining

The cells were washed by normal saline and then fixed with 4% neutral formaldehyde for 20 minutes at 4°C.

Treated with 0.2% Triton X-100 in PBS for 15 minutes, the cells were immersed into 100 mL equilibration buffer at room temperature for 15 minutes. Then, the cells were incubated with 50 μL TdT. The slides were stained by propidium iodide. Green fluorescence of apoptotic cells were detected by fluorescence microscopy.

### 2.6 Statistical analysis

Experiments were carried out at least 3 times and quantitative data were expressed as a mean ± standard deviation (SD). The differences between group were analyzed using a *t* test and 1-way analysis of variance (ANOVA) with *P* < 0.05 considered statistically significant.

## 3 Results

### 3.1 Expression of miR-34a in podocytes under HG condition

**Figure 1:**
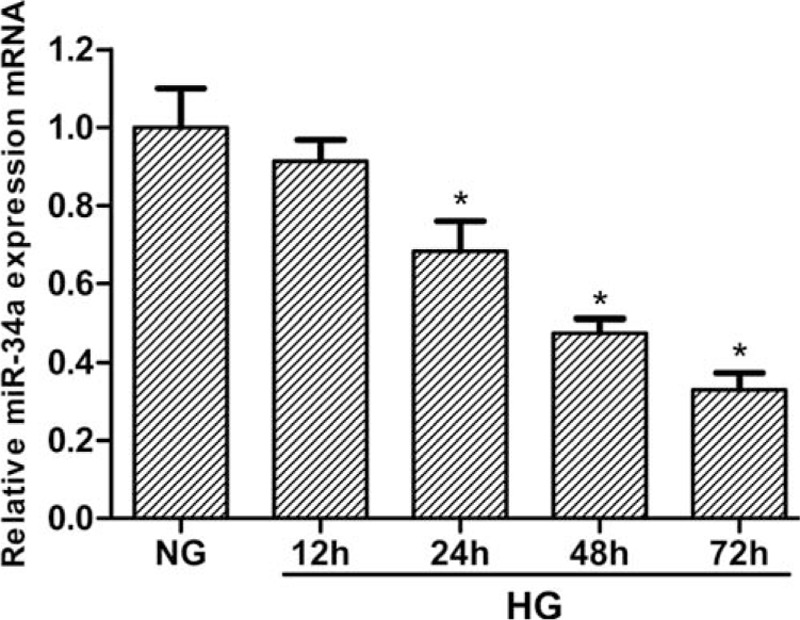
Expression of miR-34a in podocytes in the HG condition. Podocytes were incubated with 25 mM glucose (HG) at the indicated times (0–72 h). The expression of miR-34a mRNA was detected by RT-qPCR. Data represent means ± SD of 3 experiments. ^∗^*P* < 0.05 versus NG group. RT-qPCR = real-time quantitative polymerase chain reaction.

To explore the effect of miR-34a on the development of DN, the expression of miR-34a in podocytes was investigated. The podocytes were induced by HG (25 mM) for 12, 24, 48, and 72 hours. Figure 1 shows that miR-34a mRNA expression was significantly downregulated in podocytes under HG condition, suggesting the decreased level of miR-34a mRNA induced by HG was time-dependent.

### 3.2 Transfection efficiency of miR-34a minics

**Figure 2:**
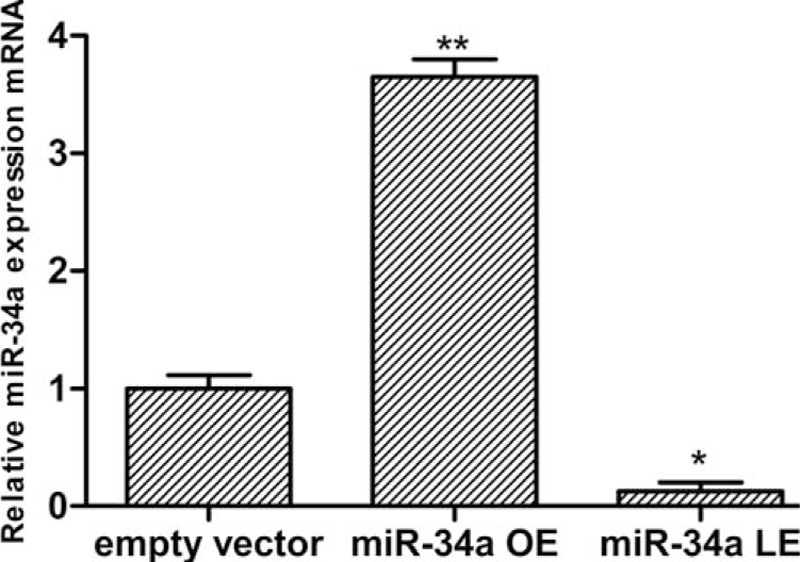
Determination of transfection efficiency in control empty vector group, miR-34a overexpression, and lower-expression group by RT-qPCR. Data represent means ± SD of three experiments. ^∗^*P* < 0.01 versus empty vector group. miR-34a OE represents miR-34a overexpression group. RT-qPCR = real-time quantitative polymerase chain reaction.

In order to measure the transfection efficiency in the EVG and miR-34a overexpression group, the relative expression of miR-34a by real-time quantitative polymerase chain reaction (RT-qPCR) was tested. Podocytes were transfected with empty vector or miR-34a minics for 24 hours. Figure 2 shows that miR-34a mRNA expression was significantly higher than EVG.

### 3.3 Relative protein expression of Notch pathway

**Figure 3:**
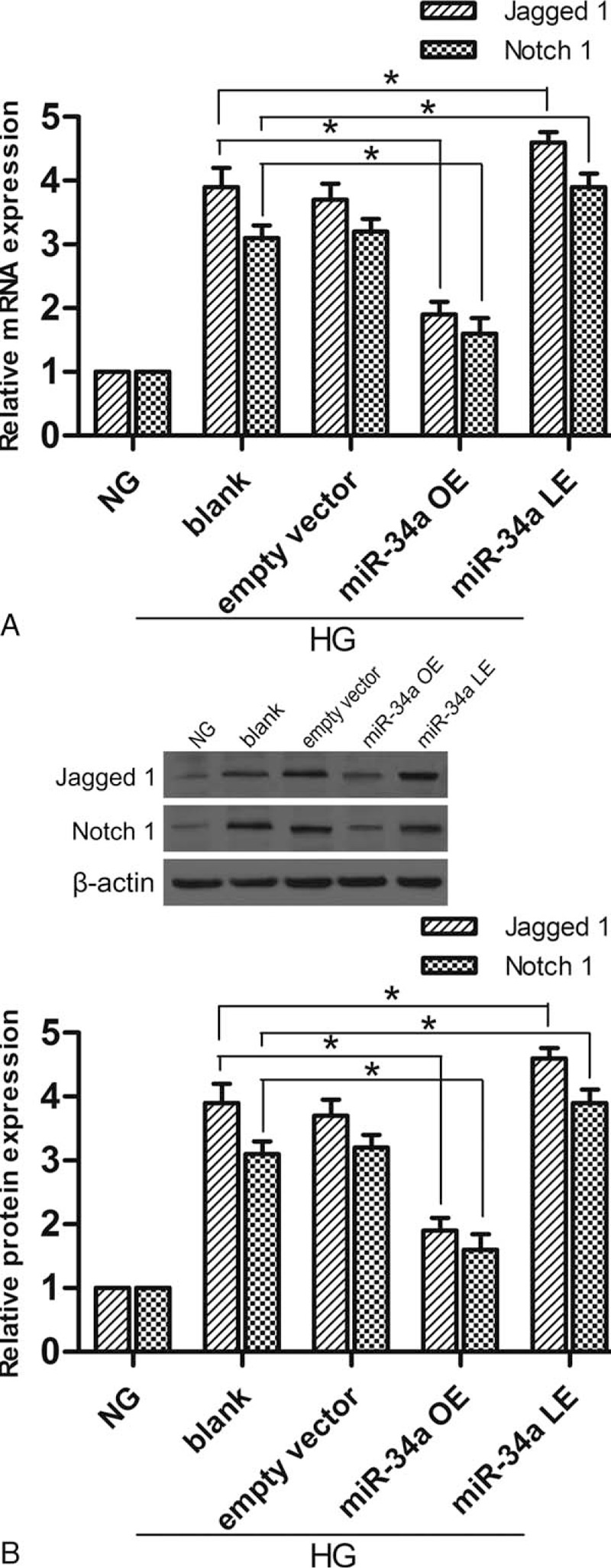
Overexpression and lower expression of miR-34a influence mRNA and protein expression of Notch 1 and Jagged 1. (A) The expression of Notch 1 and Jagged 1 mRNA was detected by RT-qPCR. (B) The expression of Notch 1 and Jagged 1 protein was analyzed by western blotting. Data represent means ± SD of 3 experiments. ^∗^*P* < 0.05 versus blank group. RT-qPCR = real-time quantitative polymerase chain reaction.

Overexpression and lower expression of miR-34a influenced the expression of related Notch pathway proteins. The protein and mRNA expression of Notch 1 and Jagged 1 was detected with western blotting and RT-qPCR respectively. Figure 3A shows that overexpression of miR-34a significantly decreased mRNA expression of Notch 1 and Jagged 1. Conversely, lower expression of miR-34a increased mRNA expression. Figure 3B shows miR-34a overexpression and lower expression in podocytes affected Notch 1 and Jagged 1 protein expression in accordance with mRNA. These results suggest that miR-34a overexpression and lower expression affect Notch pathway proteins directly.

### 3.4 The activation of Notch signaling pathway in high-glucose conditions

**Figure 4:**
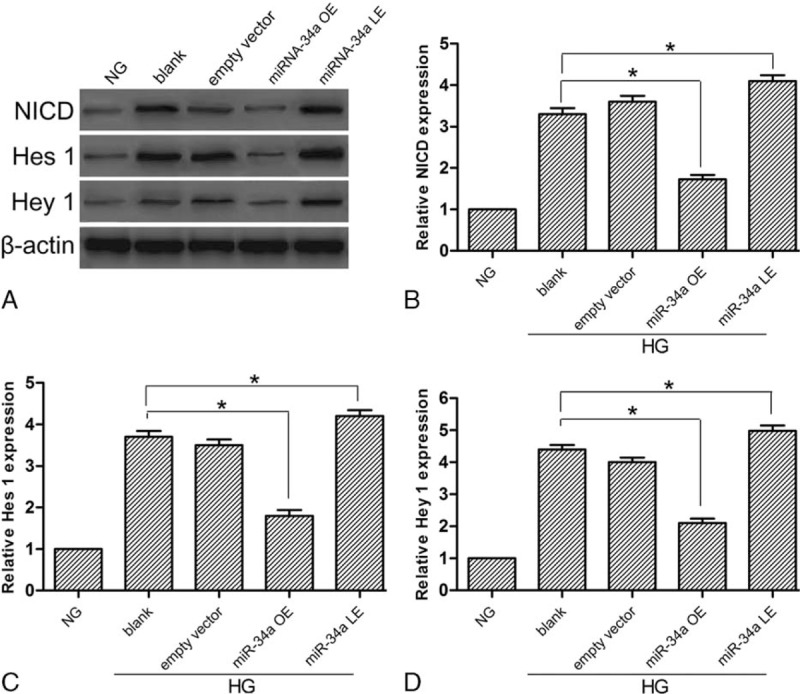
Protein expression was detected by western blotting. The expression of NICD, Hes 1, and Hey 1 increased, showing that the Notch signaling pathway was activated in HG conditions. Overexpression of miR-34a inhibited the pathway activation, while lower expression of miR-34a promoted the activation. (A) The expression of NICD, Hes 1, and Hey 1 protein was analyzed by western blotting. (B) Protein levels of NICD. (C) Protein levels of Hes 1. (D) The quantitative protein levels of Hey 1. Data represent means ± SD of 3 experiments. ^∗^*P* < 0.05 versus blank group.

The protein expression of NICD, Hes 1, and Hey 1 were examined by western blotting. NICD is the activated form of the Notch signaling pathway. Hes 1 and Hey 1 are downstream genes of the Notch signaling pathway. Figure 4 shows the expression of NICD, Hes 1, and Hey 1 increased markedly in the blank and EVG, indicating that the Notch signaling pathway was activated in HG conditions. However, the expression of NICD, Hes 1, and Hey 1 decreased in miR-34a overexpression group, indicating that miR-34a overexpression suppressed the Notch signaling pathway in HG conditions. Conversely, miR-34a lower expression upregulated the protein expression.

### 3.5 MiR-34a regulate the apoptosis of podocytes

**Figure 5:**
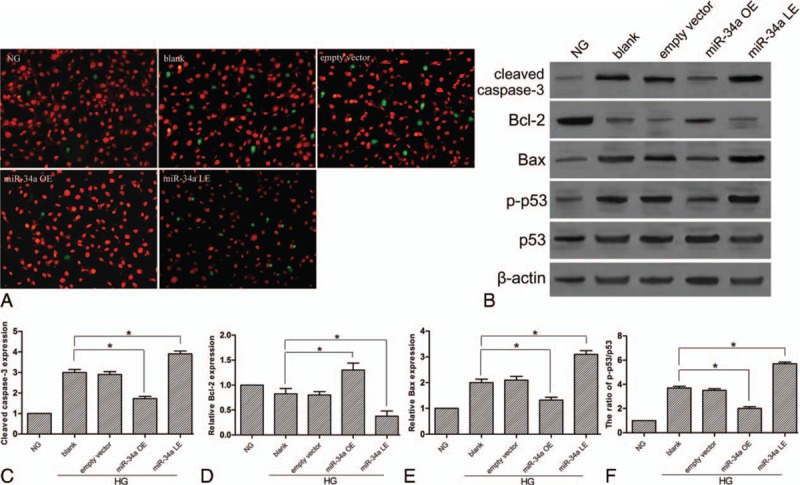
The regulation of miR-34a on podocyte apoptosis. (A) TUNEL staining of apoptotic podocytes (×200). (B) Expression levels in different groups were detected by western blotting analysis. (C) Quantitative protein levels of cleaved Casapse-3. (D) Quantitative protein levels of Bcl-2. (E) Quantitative protein levels of Bax. (F) The ratio of p-p53/p53. ^∗^*P* < 0.05 versus NG group.

In order to explore the regulation of miR-34a overexpression and lower expression of the podocyte apoptosis, we applied TUNEL staining to detect the apoptotic podocytes. Besides, the protein expression level of cleaved caspase-3, Bcl-2, and Bax were measured by western blotting. Similarly, phosphorylation activity of p53 (p-p53) was also detected. Figure 5 shows that HG conditions increased the apoptosis of podocytes and the expression of cleaved caspase-3, Bax, and p-p53, and decreased Bcl-2 expression. In miR-34a overexpression group, the apoptotic podocytes decreased and the expression of cleaved caspase-3, Bax, and p-p53 was significantly reduced. However, the opposite result was displayed in the lower-expression group. The expression of p53 remained consistent. These results indicate that miR-34a overexpression suppressed the apoptosis of podocytes.

### 3.6 MiR-34a regulate the β-arrestin expression

**Figure 6:**
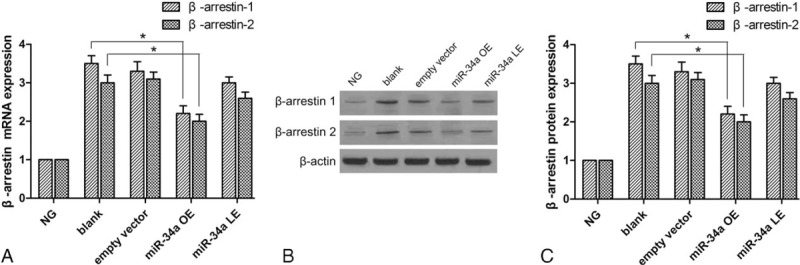
The expression of β-arrestin was detected by RT-qPCR and western blotting. (A) The expression of β-arrestin-1 and β-arrestin-2 mRNA was detected by RT-qPCR. (B, C) The expression of β-arrestin-1 and β-arrestin-2 protein was analyzed by western blotting. ^∗^*P* < 0.05 versus NG group. RT-qPCR = real-time quantitative polymerase chain reaction.

The volatility of miRNA-34a affected the expression of β-arrestin. Figure 6 shows that the mRNA and β-arrestin protein were detected by RT-qPCR and western blotting. The expression of β-arrestin-1 and β-arrestin-2 was slightly increased in the HG condition. Overexpression of miRNA-34a resulted in a partial reduction of β-arrestin.

## 4 Discussion

Numerous studies demonstrate that miR-34 family plays a significant role in tumor development,^[11]^ cardiovascular disease,^[12]^ aging,^[13]^ and kidney fibrosis.^[14]^ In recent years, miR-34a has been a focus of study in tumor-suppression. The Notch signaling pathway in podocytes regulates the development of glomerular disease.^[15]^ Building on previous research, our study explores the regulation of the Notch signaling pathway and podocyte lesions by miR-34a in vitro.

We established a podocyte DN model in HG conditions in vitro. Previous studies have demonstrated that miRNA-34 directly interact with the 3′-UTR of Notch 1 and Jagged 1, which are critical for Notch signaling pathway activation.^[16,17]^ Our results show the protein and mRNA expression of important Notch signaling pathway members (including Jagged 1, Notch 1, NICD 1, Hes 1, and Hey 1) are higher than the control group. In the pathological process of DN, high blood glucose and hemodynamic changes induce the increased expression of Jagged 1 ligand and Notch 1 receptor of the Notch signaling pathway resulting in a change in the receptor structure. γ-secretase induces the podocyte to release the NICD 1, the active form of Notch pathway, and activate the downstream genes *Hes 1* and *Hey 1*. HG and hemodynamic changes in this early stage may be responsible for the activation of the Notch signaling pathway.^[18]^ HG stimulates the glomerular mesangial cells to produce large amounts of transforming growth factor-β1 (TGF-β1) and reduce the degradation of EMC at the same time, causing glomerular sclerosis.^[19,20]^ Zhang et al^[21]^ reported that miR-34a regulated mesangial proliferation and glomerular hypertrophy by directly inhibiting GAS1 in early DN. Liu et al^[22]^ reported that miR-34c overexpression inhibits the Notch signaling pathway by targeting Notch 1 and Jaggged 1 in HG-treated podocytes.

A more recent study has demonstrated that the miR-34 family plays a significant role in the p53 regulation pathways.^[23,24]^ The transcription of miR-34s was regulated by p53 protein and was involved in the p53 pathway. DN can activate cell apoptosis related proteins (Bcl-2, p53, nuclear factor-κB). The increased ratio of Bax/Bcl-2 cause mitochondria to release cytochrome C and mediate apoptosis through a caspase cascade reaction.^[25]^ Lee et al^[26]^ found that the Bcl-2 ratio and cleaved Caspase-3 increased in podocytes and cell apoptosis was accelerated. Vogt et al^[27]^ reported the mutual exclusiveness of miR-34a methylation and p53 mutation indicates that miR-34a inactivation may substitute for loss of p53 function in cancer. Gao et al^[28]^ reported the expression of Notch 1, jagged 1, NCID, Hes 1, and Hey 1 were upregulated in podocytes in the HG conditions and mediated the apoptosis of podocytes via Bcl-2 and p53 pathways.

β-arrestins are well known for negative regulating G-protein coupled receptors (GPCRs) signaling and that they participate in receptor desensitization and internalization. In our study, β-arrestin-1 and β-arrestin-2 was slightly elevated in HG. In miR-34a overexpression group, both β-arrestin expression decreased accordingly. Conversely, the expression was higher in the miR-34a lower-expression group. Luan et al^[29]^ found that loss or dysfunction of β-arrestin-2 results in deficiency of this signal complex and disturbance of insulin signaling in vivo, thereby contributing to the development of insulin resistance and progression of type 2 diabetes. Lymperopoulos and Negussie^[30]^ reported that both β-arrestin isoforms can desensitize and internalize GPCRs, they play quite different (even opposing in certain instances) roles in the G protein independent signaling pathways. Furthermore, results suggest that β-arrestin-2 is involved in the regulation of podocyte lesions, which requires more research.

The present study suggests that the activation of the Notch signaling pathway is correlated with glomerular podocyte lesions. MiR-34a overexpression could inhibit the activation of the Notch signaling pathway in glomerular podocyte and reduce podocyte damage and ECM deposition. Therefore, miR-34a could be a novel and effective therapeutic target for the treatment of DN.

## Acknowledgment

We thank the basic medical research center of Tianjin medical university for providing technical assistance.
